# In Vitro Study of Probiotic Properties and Safety Aspects of *Saccharomyces* Yeast Strains Isolated from Traditional Fermented Food in Algeria

**DOI:** 10.3390/molecules31142440

**Published:** 2026-07-12

**Authors:** Ahmed Ararem, Adam Staniszewski, Abderrahmane Houicher, Monika Kordowska-Wiater

**Affiliations:** 1 Department of Agricultural Sciences, Faculty of Science, Amar Telidji University, BP 37 G, Laghouat 03000, Algeria; a.ararem@lagh-univ.dz (A.A.); a.houicher@lagh-univ.dz (A.H.); 2 Laboratoire de Biologie des Systèmes Microbiens, Ecole Normale Supérieure de Kouba, Algiers 16308, Algeria; 3 Department of Invertebrate Ecophysiology and Experimental Biology, University of Life Sciences in Lublin, Doświadczalna 50a, 20-280 Lublin, Poland; adam.staniszewski@up.edu.pl; 4 Department of Biotechnology, Microbiology and Human Nutrition, University of Life Sciences in Lublin, Skromna 8, 20-704 Lublin, Poland

**Keywords:** *Saccharomyces*, fermented food, probiotic, enzymatic activity, safety aspect

## Abstract

Algerian fermented foods represent a precious source of indigenous yeasts with potentially probiotic properties, which can serve as regional, functional starter cultures. The aim of the present research is to isolate and genetically identify strains obtained from traditional fermented foods and investigate their probiotic properties and safety aspects in vitro. The molecular identification revealed fifteen *S. cerevisiae* strains obtained from sourdoughs and marinated peppers samples. All tested strains showed the ability to grow at 37 °C and to survive well at 0.3% (*w/v*) bile salts with high resistance to pH 2.5. The auto-aggregation rates of tested strains reached 93.90% after 24 h of incubation, while seven of fifteen strains showed high hydrophobicity values in xylene (44.60–58.90%), indicating their ability to survive and to adhere to the host intestinal mucosa. All *S. cerevisiae* isolates showed a good antioxidant activity and exhibited varying levels of antibacterial activity, but not against *Listeria innocua.* A lack of hemolysis, gelatinase, protease and phospholipase was recorded for all tested strains, while a strong phytase activity was observed in these strains, which may enhance mineral availability and reduce antinutritional effects of plant-based food. Based on these results, five *S. cerevisiae* strains showed promising probiotic properties and safety aspects, which can be used as potential probiotic strains in functional food industries and/or in the medical field.

## 1. Introduction

Algeria, a Mediterranean country, has a rich and diverse tradition in food technology, and many traditional foods of plant or animal origin are highly appreciated and widely consumed. In many rural regions, spontaneous fermentations are still the main method for traditional preservation of food products. This process improves the development of desirable characteristics and organoleptic properties, ensures microbial stability and safety, and extends shelf life. Several yeasts and/or bacteria involved in natural fermentations of many traditional foods such as meat and dairy products, cereal-based foods, and marinated vegetables ensure the development of the final products [1,2]. Yeast can possess traits that exhibit health benefits for the consumer as a probiotic and play several important roles during natural fermentation including improvement of texture, aroma, and flavor, reduction in toxins and antinutritional factors, development of nutritional properties and production of alcohol and bioactive components, acting in synergy with other groups of microorganisms belonging to lactic acid bacteria and filamentous fungi [3].

The genus *Saccharomyces* includes *Saccharomyces cerevisiae*, a well-known yeast, used for centuries in fermentation processes and industrial baking. It also has diverse applications in biotechnology, bioremediation and bioenergy, as well as in medical field as a probiotic agent for preventing or treating various infections and disorders [4,5]. *Saccharomyces cerevisiae* var. *boulardii* is the most prominent probiotic yeast used in many countries as a biotherapeutic agent for the prevention and treatment of several intestinal disorders and infections, including inflammatory bowel disease and *Clostridium difficile* intestinal infections [6]. In fact, good survival at low pH and physiological bile concentration, high aggregation abilities and cell-surface hydrophobicity, biofilm formation, antimicrobial effects, anti-toxigenic and phytate-degrading capabilities, and high adaptability to stressful niches, as well as health-promoting effects are interesting criteria of the probiotic yeasts [3,5,7].

Algerian fermented foods represent a precious source of indigenous yeasts with several health benefits for the consumer of the product, reflecting the microbiota of the geographical area where they have been produced. However, only few reports are available on the isolation and characterization of probiotic yeasts, especially *Saccharomyces* spp. from this variety of sources which remains incompletely explored. Moreover, isolation and identification of yeasts belonging to the genus *Saccharomyces* from this geographical area may yield fruitful information about *Saccharomyces* species distribution and diversity in traditional foods, and also about the phenotypic variations in *Saccharomyces* strains involved in natural fermentations for future application in functional food industries as potential probiotics. In addition, several previous studies showed that many species of the genus of *Saccharomyces* can possess probiotic properties, in which the selection of new probiotic strains with novel properties from traditional foods has given a particular interest [2,6,8].

Given these considerations, this research specifically aims to: (1) genetically isolate and identify the different *Saccharomyces* strains obtained from traditional fermented foods in the Laghouat region of Algeria; (2) investigate the probiotic properties of *Saccharomyces* spp. isolates, including auto-aggregation and hydrophobicity of the cell surface, survival ability under bile salts and acidic pH conditions, antioxidant and antibacterial activity, and safety aspects and enzymatic activities in order to select potential candidate strains for future application as probiotics in food industries and/or in the medical field.

## 2. Results

### 2.1. Strains Selection and Identification

**Figure 1:**
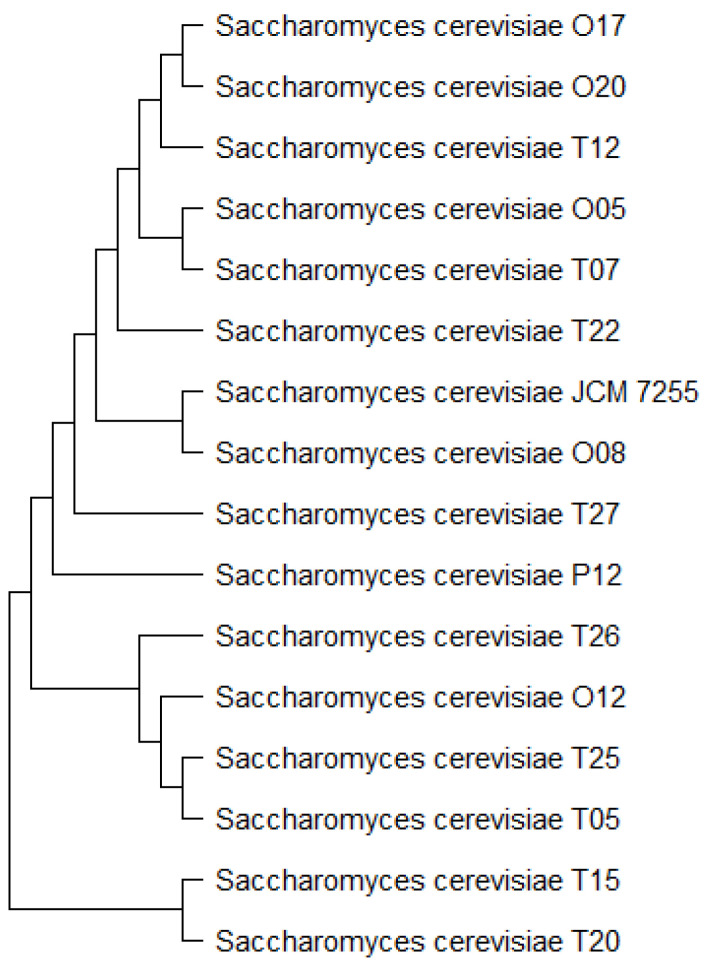
Phylogenetic tree of *Saccharomyces cerevisiae* strains, based on D1/D2 domain of 28S rDNA region, including the reference strain *S. cerevisiae* JCM 7255 (accession number U44806).

In this study, a total of 53 yeasts were isolated from fermented food samples. Based on their colony characteristics on WL medium (cream to green knoblike appearance, smooth and opaque surface) and microscopic examination (ovoid cell shape, presence of ascospores, multipolar budding pattern), as well as the absence of lactose fermentation and assimilation, 15 isolates were selected for molecular identification. As shown in Table 1, the genotypic identification revealed that all isolates obtained from sourdoughs and marinated pepper samples belonged to the same species—*Saccharomyces cerevisiae*. The results of the phylogenetic analysis based on the sequence of the D1/D2 domain of a large subunit rRNA gene are presented in Figure 1. However, no *Saccharomyces* strain was isolated from Lben and marinated olives samples.

In addition, all tested *Saccharomyces cerevisiae* strains showed the ability to utilize sucrose, maltose, and glucose except for *S. cerevisiae* P12. For galactose, its profile was highly variable within strains. None of the strains utilize lactose, arabinose, and xylose. Notably, *S. cerevisiae* P12 only grew poorly on glucose alone and failed to grow on other tested sugars (Table 2).

### 2.2. Probiotic Properties of Saccharomyces Strains

#### 2.2.1. Growth at 37 °C

All fifteen *S. cerevisiae* strains showed the ability to grow at 37 °C, which is the basic selection criterion for further analyses.

#### 2.2.2. Bile Salts and Acidic pH Tolerance

**Figure 2:**
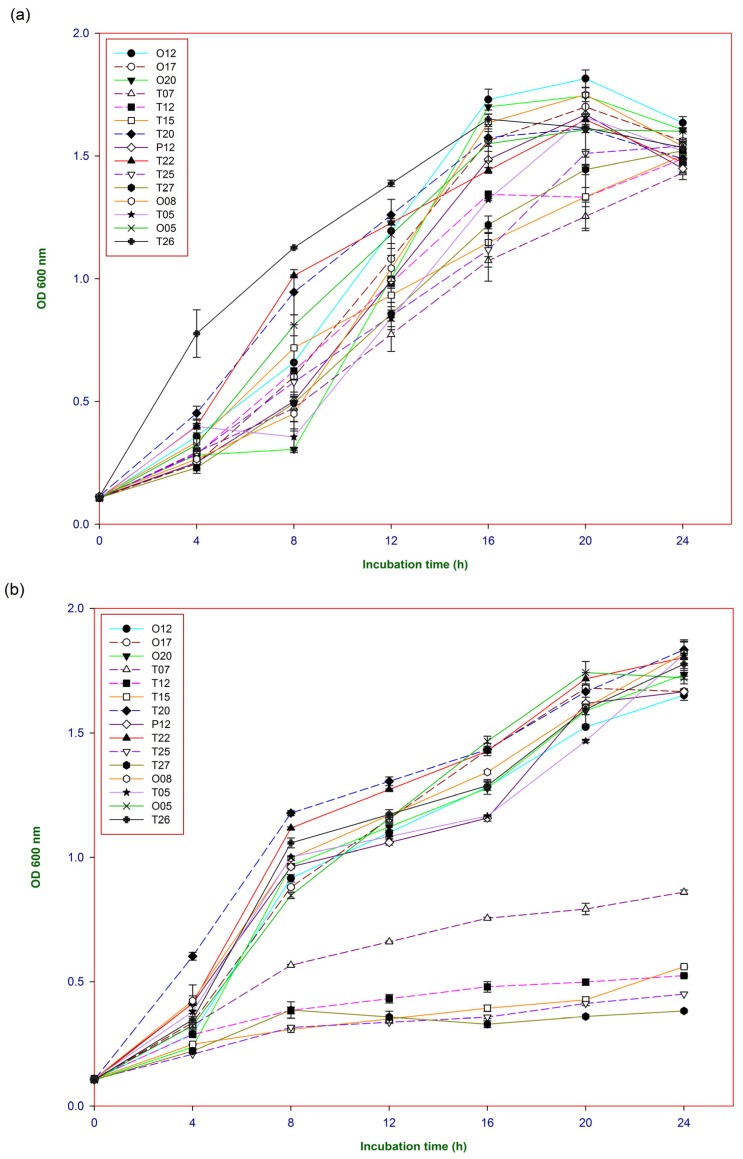
Gastrointestinal stress tolerance of *Saccharomyces cerevisiae* strains: (**a**) growth in presence of 0.3% bile salts; (**b**) growth under acidic conditions (pH 2.5) at 37 °C. Standard deviations are indicated by bars (*n* = 3).

With a slight variation depending on the strains, all isolates of *S. cerevisiae* grew well at 0.3% bile salts, which revealed increased growth after 8 h of incubation at 37 °C (Figure 2a and Table S1). In addition, the tested strains showed high resistance to pH 2.5 until 4 h of incubation and then their growth significantly increased, reaching the levels of 7.5 log CFU/mL after 24 h of incubation at 37 °C, except for five strains that showed a slight increase (Figure 2b and Table S2). Our findings indicate that all tested strains showed good tolerance to gastrointestinal stress and demonstrated their potential to survive under these conditions, as they can be used as potential probiotic strains.

#### 2.2.3. Auto-aggregation Assay

**Figure 3:**
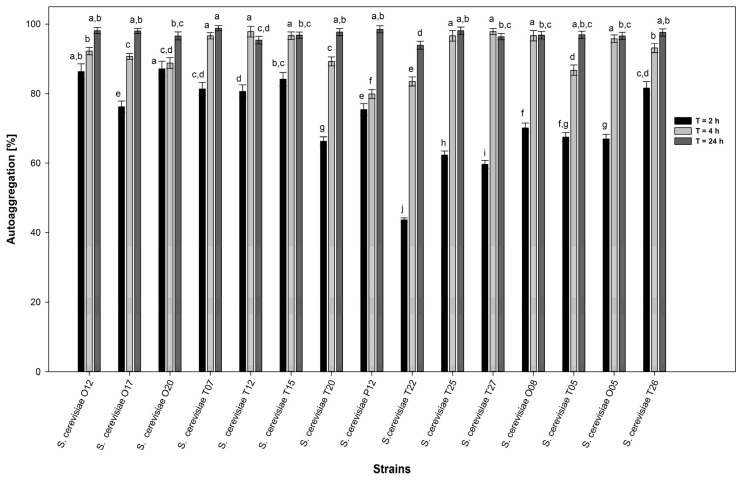
Auto-aggregation rates (%) of *S. cerevisiae* strains over 2, 4, and 24 h. Standard deviations are indicated by bars (*n* = 3). Different lowercase letters (a–j) in the same incubation time indicate significant differences (*p* < 0.05) among strains.

After two hours of incubation at 37 °C, all *S. cerevisiae* strains exhibited auto-aggregation levels above 60%, with the exception of *S. cerevisiae* strains T22 (43.66%) and T27 (59.66%). As shown in Figure 3, the auto-aggregation rates of all strains remarkably increased with incubation time, exceeding the level of 93.90% after 24 h of incubation at 37 °C. The highest auto-aggregation value was recorded for *S. cerevisiae* T07 (98.80%), while the *S. cerevisiae* T22 strain showed the lowest auto-aggregation value (93.90%). Notably, all strains showed high auto-aggregation ability, which could be favorable for adhering to epithelial cells and exerting their beneficial effects.

#### 2.2.4. Cell Surface Hydrophobicity

**Figure 4:**
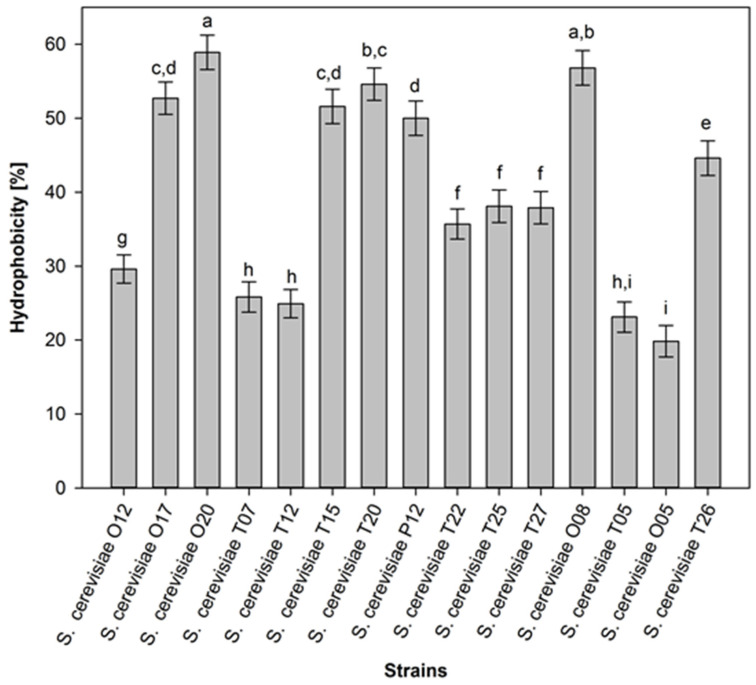
Cell surface hydrophobicity percentages of *S. cerevisiae* isolates. Standard deviations are indicated by bars (*n* = 3). Different lowercase letters (a–i) indicate significant differences (*p* < 0.05) among strains.

In our study, seven of fifteen strains showed high hydrophobicity values in xylene, ranging from 44.60% to 58.90%, indicating their potential to survive and to adhere to the host intestinal mucosa (Figure 4). The other strains exhibited low hydrophobicity levels, less than 40%, in which the lowest hydrophobicity percentage was recorded for two strains, *S. cerevisiae* T05 (23.10%) and *S. cerevisiae* O05 (19.83%).

#### 2.2.5. Antioxidant Activity

**Figure 5:**
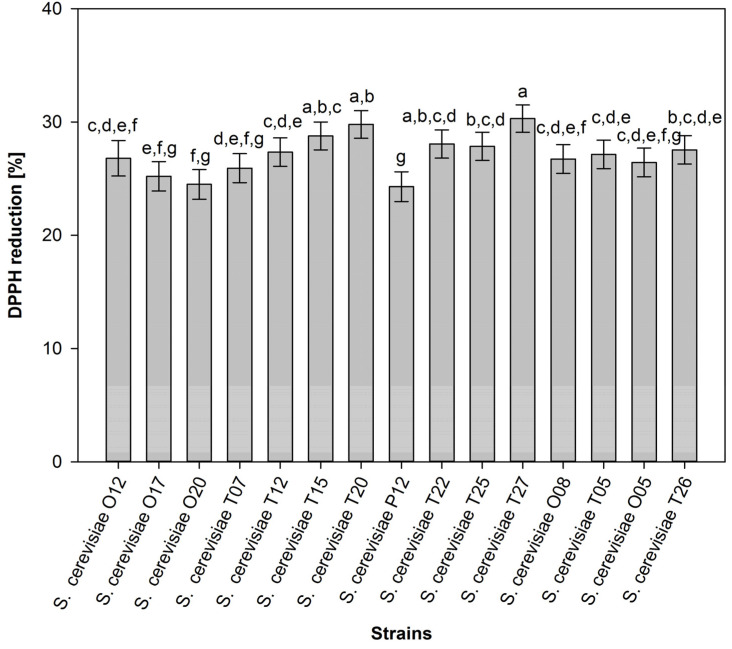
DPPH reduction percentages of *S. cerevisiae* isolates. Standard deviations are indicated by bars (*n* = 3). Different lowercase letters (a–g) indicate significant differences (*p* < 0.05) among strains.

With slight variation among *S. cerevisiae* strains, all isolates exhibited a notable antioxidant potential through the DPPH free radical scavenging activity (Figure 5). The Antioxidant assay showed effective DPPH reduction percentages, ranging from 24.29% to 30.29% for *S. cerevisiae* P12 and *S. cerevisiae* T27, respectively. In addition, a positive catalase reaction was also detected in all strains, indicating their potential to reduce oxidative stress and to prevent degenerative diseases and/or disorders.

#### 2.2.6. Antibacterial Activity

Table 3 summarizes the antibacterial activity of *S. cerevisiae* strains against pathogenic bacteria surrogates. All *S. cerevisiae* strains did not exert any activity toward *Listeria innocua*. With the exception of *S. cerevisiae* T22 and *S. cerevisiae* T26, tested strains showed a weak activity against *Staphylococcus aureus*, in which three strains only, *S. cerevisiae* O17, O08, and O05, indicated a moderate inhibitory effect. In terms of *Escherichia coli*, four *S. cerevisiae* strains showed a moderate antibacterial activity, while a weak inhibition was observed for the other strains against this bacterium, except for *S. cerevisiae* O17, T25, and O08 (no inhibition zone).

#### 2.2.7. Enzymatic Activities and Safety Aspects

Among the enzymatic activities, phytase, protease, phospholipase, and gelatinase production were evaluated (Table 4).

All *S. cerevisiae* strains showed a strong phytase activity, characterized by the formation of large and clear zones. This suggests a strong capacity for phytate degradation, which may improve the bioavailability of minerals for absorption and enhance the nutritional value of fermented plant-based products. However, the tested strains showed negative reactions for protease and phospholipase activities by the absence of clear zones and precipitation areas around colonies, respectively.

In this study, no hemolytic activity was detected in all *Saccharomyces* strains, which is considered as γ hemolysis, and none of these strains produced gelatinase, confirming their safety to be used as probiotics (Table 4). In addition, all *S. cerevisiae* strains proved their safety by showing a negative biogenic amine production, except for the strain *S. cerevisiae* T27, which showed a purple halo around the colony area demonstrating the ability to produce one or more biogenic amines.

## 3. Discussion

Yeast strains isolated from traditional Algerian fermented food were identified via D1/D2 domain analysis and belong to *Saccharomyces cerevisiae* [9,10,11,12]. This analysis is routinely used in ascomycetous yeast systematic [13]. The results are consistent with the results obtained by Bazalová et al. [14], where yeasts isolated from different kinds of sourdough include *S. cerevisiae* as the most frequently occurring yeast species. Palla et al. [15] investigated yeast populations of traditional sourdoughs collected from four Tuscan bakeries and among 78 randomly selected and molecularly characterized isolates, *S. cerevisiae* represented the only species detected in three out of the four sourdoughs. In turn, in Vrancken et al.’s [16] research, 68% of yeasts isolated from 127 artisan sourdoughs were genetically identified as *S. cerevisiae*, including by the D1/D2 LSU-PCR method. Similarly, Bruner and Fox [17] reported that different *Saccharomyces* species were shown to ferment sucrose, maltose, and glucose, but did not ferment lactose, which are common characteristics of *Saccharomyces* sensu stricto yeasts.

In this study, all tested strains showed the ability to grow at 37 °C and to survive well at 0.3% (*w*/*v*) bile salts with high resistance to pH 2.5. The selection of yeast strains with high adaptation to corporal temperature (37 °C) is considered a key criterion for probiotic strains selection, reflecting their potential to exert beneficial effects on the human body [18]. Moreover, the ability of strains to resist physiological bile salt concentrations and low pH is an important criterion for selecting probiotic strains that enhance their capacity to benefit the host by continuing to colonize the gastrointestinal tract, maintaining their cellular integrity and retaining their beneficial metabolic functions [19,20]. Tolerance to bile salts is an important criterion for the selection of probiotics and the small intestine contains relatively high concentrations of bile salts, varying from 0.2% to 2.0%, which is deleterious for living cells. Apart from the bile salts, acidic pH tolerance (pH 2.0–2.5) is also considered a key requirement for selecting probiotic strains [21]. Similarly, several in vitro studies indicate that yeasts belonging to the *Saccharomyces* genera are tolerant to high concentrations of bile salts and stomach-acidic pH [18,21,22].

The ability of probiotic strains to form cell aggregates is associated with adherence to epithelial cells of the intestinal mucosa, enabling their colonization in the gastrointestinal tract before exerting their beneficial effects [23]. Yeast cells are heavier and larger than that of bacteria, allowing them to precipitate quicker and in higher proportions [22]. The strain-specific composition of the cell wall, including the presence of surface appendages or protruding macromolecules, affects auto-aggregation, which is mainly mediated by molecules on the cell surface [24]. The variability among the tested strains in our study may be attributed to these structural variations.

Hydrophobicity is a crucial functional trait that facilitates the adhesion of cells to the gastrointestinal tract, thus enhancing their interaction with intestinal tissue, exerting their health effects, and preventing intestinal disorders [25,26]. Yeast strains showing hydrophobicity levels higher than 40% may be considered hydrophobic [27]. Romero-Luna et al. [23] and Pereira et al. [21] reported that *S. cerevisiae* strains showed higher hydrophobicity for chloroform than for hexadecane and o-xylene. In addition, hydrophobicity is a complex multistep process and involves both hydrophobic forces and electrostatic interactions between hydrophilic and hydrophobic components on the surface of microbial cells and intestinal mucosa [18,22]. The high hydrophobicity observed in *S. cerevisiae* strains supports their ability to colonize the gastrointestinal tract and shows their probiotic potential.

The consumption of probiotic microorganisms or foods possessing antioxidant properties reduces free radical generations and prevents degenerative diseases and/or disorders, such as cell apoptosis, autoimmune disorders and cancer [21]. Several authors indicate that the intact yeasts had higher antioxidant activities than the extracts, which may be attributed to certain active compounds produced on their cell wall, such as superoxide dismutase, glutathione peroxidase, catalase, (1/3)-β-D-glucan and other β-glucans [18,22,28]. In this study, all *S. cerevisiae* strains showed good antioxidant activity with DPPH reduction percentages, ranging from 24.29% to 30.29%, which remains higher than those reported by Menezes et al. [18], Hsiung et al. [22], and Goktas et al. [28]. However, percentages of DPPH free radical scavenging activity vary from study to study and from strain to strain, and these variations may be due to different experimental conditions, mainly yeast concentration, intact or extract cell, and incubation time, as well as DPPH preparation and concentrations. In addition, all strains showed the ability to transform reactive oxygen species of hydrogen peroxide into O_2_ and H_2_O as part of an enzymatic defense system, exhibiting positive catalase activity; this can effectively contribute to reducing inflammation in patients with inflammatory bowel disease, as reported by Fernández-Pacheco et al. [29].

The inhibitory activity of yeasts against pathogenic bacteria contributes to the safety of fermented foods and plays a key role in preserving their sensory quality through the production of antimicrobial metabolites [30]. So, the tested *S. cerevisiae* strains exhibited varying levels of antibacterial activity but none of the strains inhibited the growth of *L. innocua*, which suggests that this bacterium species may not be as sensitive to the antimicrobial compounds produced by *Saccharomyces* yeasts. Similarly, Fakruddin et al. [31] reported that a *S. cerevisiae* isolate showed better antibacterial activity against Gram-negative bacteria than Gram-positive ones, but none of the *S. cerevisiae* strains isolated by Zeng et al. [32] and Fernandez-Pacheco et al. [30] were able to inhibit the growth of pathogens. Our findings fit with what has been found by Fernandez-Pacheco et al. [30] which stresses that yeast-mediated inhibition is strain- and target-specific. In general, most *S. cerevisiae* strains showed a weak inhibition toward *S. aureus*, whereas three tested strains exerted moderate antibacterial activities. These results suggest that some *Saccharomyces* strains may be able to synthesize antimicrobial metabolites or use competitive exclusion mechanisms that work against Gram-positive bacteria. *Saccharomyces* yeasts can produce several antimicrobial metabolites, including volatile organic compounds, organic acids, killer toxins, and ethanol, which may inhibit pathogenic bacteria by lowering environmental pH, disrupting cell membranes, or interfering with bacterial metabolism [6,31]. On the other hand, only four of the tested *Saccharomyces* strains showed a moderate inhibition against *E. coli*, a Gram-negative bacterium, which is typically more resistant to antimicrobial action, while the majority of the remaining strains displayed only weak inhibitions. In agreement with the findings of Staniszewski et al. [33], our results demonstrate the diversity in the antimicrobial effects of yeasts and validate that such activity is strain-specific. They also suggest that some *Saccharomyces* strains can bind, degrade, or neutralize bacterial toxins, particularly toxins synthesized by *S. aureus* and *E. coli*, which may reduce their harmful effects on host tissues and limit pathogen virulence [34]. These results highlight the potential application of some *Saccharomyces* strains in enhancing food safety through targeted antibacterial action.

Phytase is considered effective at reducing antinutritional effects and enhancing mineral availability in fermented foods by breaking down phytate, thereby improving the nutritional and functional value of proteins [35]. Similarly to the finding of Lama et al. [36], all tested strains demonstrated the ability to hydrolyze phytate, supporting their role as beneficial strains for enhancing nutrient availability and food functionality. In addition, a lack of protease and phospholipase productions may have been caused by environmental restrictions or enzyme regulation. In agreement with our findings, Pereira et al. [21] reported that the results were negative for protease and lipase and none of the *S. cerevisiae* strains exhibited the production of the extracellular enzymes tested. Protease and phospholipase production may contribute to pathogenicity when they are in high concentrations by increasing the ability of certain microorganisms to penetrate and colonize the host tissue, breaking the membranes of the epithelial cells, and causing tissue damage in the host cells [37]. However, the ability to produce extracellular enzymes such as protease and lipase improves nutrient utilization in the intestine and provides health benefits. They are also involved in the sensory properties of foods by breaking down complex compounds during fermentation [21].

Probiotic strains must lack virulence factors, such as hemolysin and gelatinase. No hemolytic activity was detected in all tested *Saccharomyces* strains and none of these strains produced gelatinase. Hemolysis may affect the host by facilitating iron availability and causing anemia and edema [23]. Lipilkina et al. [38] pointed out the possible risks of probiotics that contain gelatinase, particularly with regard to the degradation of host tissue and heightened susceptibility to infections. Moreover, the presence of biogenic amines in food, especially histamine, can adversely affect human health, particularly in people sensitive to these compounds. Consequently, characterizing *Saccharomyces* strains for their ability to produce biogenic amines has become a major priority for protecting consumer health [36]. In this study, only one strain (*S. cerevisiae* T27) is capable of producing biogenic amines, but other strains are considered as safe and promising candidates for food additives. These results are consistent with those reported by Staniszewski and Kordowska-Wiater [39], highlighting the possibility that biogenic amine production may vary even within a single species.

## 4. Materials and Methods

### 4.1. Food Sampling

This study investigated the diversity of *Saccharomyces* spp. in some traditional fermented foods in the Laghouat region of Algeria, including sourdoughs made with soft wheat and barley flours, marinated vegetables (peppers and olives), and fermented cow milk or Lben. Sourdough fermentation was carried out using wheat and barley flours, undergoing three refreshment cycles with warm physiological water and flour (*v/w*) [40]. In addition, marinated peppers and olives were preserved in a 12% saline solution according to the traditional pickling procedure as described by Benkerroum [1], while Lben was obtained from spontaneous fermentation of cow milk, undergoing 12 to 120 h at room temperature [41].

### 4.2. Yeast Isolation and Identification

To isolate yeasts, three samples from each fermented product were taken and a saline decimal dilution series was prepared from each food sample up to 10^–4^. Then, yeasts were isolated by spreading 100 µL aliquot from each dilution onto YPD agar (1% yeast extract, 2% peptone, 2% dextrose, 1.7% agar) supplemented with 0.01% chloramphenicol [42]. After incubation of plates at 25 °C for 72 h, the resulting colonies were purified by repeated streaking on the same medium. Pure isolates were stored at 4 °C in YPD broth for short-term storage and at −80 °C in YPD broth supplemented with 20% glycerol (*v*/*v*) for long storage periods.

#### 4.2.1. Morphological Identification

For the morphological identification, pure cultures were streaked onto WL agar (Wallerstein Laboratories medium) (Wallerstein Laboratories, Oxoid, Hampshire, UK) and then the inoculated plates were subsequently incubated at 25 °C for 72 h in order to analyze the colony morphology, the pigmentation, and the topographical structure [33]. For the microscopic examination of yeasts, overnight cultures were observed using an optical microscope (Zeiss, Axiostar Plus, Oberkochen, Germany) at 400× magnification to confirm their morphologic characteristics, including cellular shape, mode of reproduction, and cellular arrangement, as described by Kurtzman et al. [7].

#### 4.2.2. Sugar Fermentation and Assimilation

To test the fermentation and assimilation of carbohydrate sources by yeast isolates, a YP broth containing 0.5% yeast extract, 0.7% peptone, 0.06% of bromocresol purple, and supplemented with 2% of the tested sugars (glucose, maltose, lactose, sucrose, galactose, xylose, and arabinose) was used [43]. After 24 h of incubation under anaerobic conditions at 37 °C, sugar fermentation was confirmed by CO_2_ production, as indicated by gas accumulation in Durham tubes, while assimilation was assessed through a visible color change of the tested medium from purple to yellow.

#### 4.2.3. Molecular Identification

The yeast isolates were incubated in liquid YPG medium at 30 °C for 2 days to obtain fresh biomass, after which the pre-ground-in-liquid-nitrogen sample was centrifuged at 8000× *g*. DNA isolation from yeast biomass was performed according to the procedure of Gene Matrix Plant & Fungi DNA Purification Kit (Eurx^®^ Ltd., Gdańsk, Poland). The identification of the yeasts’ strains was based on the amplification of the D1/D2 domain of the large subunit of rRNA (LSU) gene and sequencing amplicons using the Sanger method. Polymerase chain reaction (PCR) was carried out in FIREPol Master Mix (Solis Biodyne, Tartu, Estonia) using primers acc. to Kurtzman and Robnett [44]-NL1 (5′ GCA TAT CAA TAA GCG GAG GAA AAG-3′) and NL4 (5′-GGT CCG TGT TTC AAG ACG G-3′) (Genomed Ltd., Warsaw, Poland) to amplify the D1/D2 domain. Amplification was conducted in a thermocycler (T100^TM^ Thermal Cycler, Bio-Rad, Hercules, CA, USA) at following conditions: initial DNA denaturation at 95 °C for 3 min; 30 cycles included denaturation at 95 °C for 1 min, annealing at 58 °C for 30 sec, and extension at 72 °C for 1 min with final extension at 72 °C for 5 min. Electrophoresis in 1% (*w/v*) agarose gel in TAE buffer (Mini-Sub Cell GT and Power Pac Basic Bio-Rad, Hercules, CA, USA) was used for visualization of specific PCR product for each strain with the use of Gel Doc XR+ (Bio-Rad, Hercules, CA, USA). Purification and sequencing of the amplicons was performed by Genomed (Warsaw, Poland). Obtained sequences and contigs assembled with DNA Sequence Assembler v5 (Heracle BioSoft, Mioveni, Romania), www.DnaBaser.com (accessed on 3 February 2026) were compared to the known sequences of the D1/D2 domain of the 28 rDNA region in National Center for Biotechnology Information (NCBI) GenBank database by alignment with the BLAST algorithm https://blast.ncbi.nlm.nih.gov/Blast.cgi (accessed on 3 February 2026) and the isolates were identified on the basis of the highest value of cover and percentage identity. Evolutionary analyses were conducted in MEGA11. The phylogenetic tree was created based on the D1/D2 domain of 28 rDNA region sequences using the maximum likelihood method with the best-fit Kimura 2-parameter model with a discrete gamma distribution (+G), with 1000 bootstrap replication. The reference strain *S. cerevisiae* JCM 7255 with the accession number of LSU D1/D2 sequence U44806 was used to construct the phylogenetic tree [44,45]. The strain characteristics can be found in the JCM catalog via the link: https://www.jcm.riken.jp/cgi-bin/jcm/jcm_number?JCM=7255 (accessed on 22 June 2026).

### 4.3. Probiotic Properties and Safety Aspects

#### 4.3.1. Growth at 37 °C

For testing the growth of strains at human body temperature, 1 mL of YPD broth was inoculated by 10 µL of fresh culture and incubated at 37 °C for 2 days. The strains’ ability to grow under these conditions was determined by visual observation of the growth in assay tubes [33]. The growth of the yeast strain under such conditions is considered as the first selection criterion for subsequent analyses.

#### 4.3.2. Bile Salts and Acidic pH Tolerance

To assess the gastrointestinal stress tolerance of *Saccharomyces* strains, overnight cultures were inoculated in YPD broth as control and YPD broth either containing 0.3% bile salts (*w/v*) or adjusted to pH 2.5 using 1 mol/L HCl to obtain an optical density about 0.1 at 600 nm (OD_600_). The assay was conducted in 25 mL tubes, and the growth was measured at OD_600_ every 4 h using a Spectrophotometer (KLAB, OptizenTM pop, Daejeon, Republic of Korea) during 24 h incubation at 37 °C [46].

#### 4.3.3. Auto-aggregation Assay

The auto-aggregation of *Saccharomyces* strains was evaluated according to the method of Hossain et al. [47] and Staniszewski et al. [33]. Fresh cultures were centrifuged at 5000× *g* for 10 min, washed twice with saline solution 0.85%, and then resuspended in the same saline solution. To determine the auto-aggregation of strains, 3 mL of cell suspension was vortexed for 10 sec, and the absorbance was measured without shaking at OD_600_ using a Spectrophotometer (KLAB, OptizenTM pop, Daejeon, Republic of Korea) after 2, 4, and 24 h incubation at 37 °C.

Auto-aggregation was quantified using the following equation:

Auto-aggregation (%) = [1 − (A_t_/A_0_)] × 100

where A_t_ represents the OD_600_ at 2, 4, and 24 h, and A_0_ is the OD_600_ at time zero.

#### 4.3.4. Cell Surface Hydrophobicity

Cell surface hydrophobicity (CSH) was evaluated using the solvent-based method, as described by Staniszewski et al. [33]. To conduct the assay, 1 mL of overnight cultures was centrifuged at 5000× *g* for 10 min. The cell biomass was then washed twice with PBS (potassium phosphate buffer) at pH 7.0, and resuspended in 5 mL of the same buffer. The initial absorbance was measured at 600 nm (OD_600_) using a Spectrophotometer (KLAB, OptizenTM pop, Daejeon, Republic of Korea). After that, 1 mL of xylene was added to 3 mL of the yeast suspension, and vortexed for 2 min to facilitate the interaction with the solvent. The mixture was incubated at 37 °C for 30 min to allow the separation of phases. The aqueous phase was carefully removed, and then the OD_600_ after extraction was measured. According to Hossain et al. [47], the percentage of CSH was quantified using the formula:

Hydrophobicity (%) = [(A_initial_ − A_finale_)/A_initial_] × 100

where A_initial_ represents the OD_600_ before extraction and A_finale_ is the OD_600_ after extraction with the solvent.

#### 4.3.5. Antioxidant Activity

The antioxidant activity of the strains was studied by testing their ability to scavenge the free radical DPPH (1,1-diphenyl-2-picrylhydrazyl), as described by Trotta et al. [48]. Briefly, 1 mL of washed yeast suspensions, with a concentration ranging between 10^7^ and 10^9^ CFU/mL, was mixed with 2 mL of 0.2 mM DPPH ethanolic solution. The mixture was then incubated at 37 °C for 30 min. The absorbance was measured at 517 nm after centrifugation of the mixture for 10 min at 8000× *g*. All measurements were performed in triplicate and the percentage of DPPH scavenging activity was obtained as follows:

DPPH scavenging activity (%) = [1 − (A_517_ sample/A_517_ control)] × 100

The second test was conducted to assess catalase enzyme activity, according to Fernández-Pacheco et al. [29]. For each yeast strain, the test was done by adding 3% hydrogen peroxide (*v*/*v*) to 300 µL of a fresh suspension containing 10^6^ CFU/mL. This led to the appearance of bubbles, indicating positive activity.

#### 4.3.6. Antibacterial Activity

Antibacterial activity was assessed using the agar well diffusion method, according to the protocol of Fernandez-Pacheco et al. [30]. Suspensions of each fresh surrogate of pathogenic strain (*Staphylococcus aureus* ATCC 29213, *Escherichia coli* ATCC 25922 and *Listeria innocua* ATCC 33090) were prepared in BHI (brain heart infusion) broth and distributed onto the surface of nutrient agar plates with a final concentration of about 10^7^ CFU/mL. Wells of 5 mm diameter were cut in the solidified agar containing target pathogens using cock-borer, and then yeast suspensions (approximately 10^6^ CFU/mL) were put into wells. The plates were incubated at 30 °C for five days. The antagonist effect was demonstrated by measuring the radius of the clear halos around the wells. The diameter of the inhibition zone was measured in millimeters and classified as described by Younis et al. [49]: −, no inhibition zone ≤ 5 mm; weak inhibition zones 6–10 mm; moderate inhibition zones 11–15 mm; strong inhibition zones ≥ 16 mm.

#### 4.3.7. Enzymatic Activities and Safety Aspects

##### Phytase Production

The isolates’ phytate hydrolysis capability was assessed following the method described by Lama et al. [36]. This activity was tested on the phytase screening agar consisting (g/L): glucose 15.0; phytic acid sodium salt hydrate from rice 1.0; (NH_4_)_2_SO_4_ 5.0; MgSO_4_.7H_2_O 0.5; KCl 0.5; FeSO_4_. 7H_2_O 0.01; MnSO_4_. 1H_2_O 0.1; and agar 15.0. The pH was adjusted to 5.5 with 0.1 N NaOH. The plates were incubated at 37 °C for 24 h, during which a clear zone around the colonies indicated phytase production.

##### Proteolytic and Lipolytic Activity

According to the protocol of Zeng et al. [32], protease activity was assessed on a skim milk agar medium (0.25% yeast extract, 0.5% peptone, 0.1% glucose, 1% skim milk, 1.5% agar). The tested medium was then inoculated by fresh cultures and incubated for three days at 37 °C. Colonies exhibiting a clear halo confirmed positive protease activity and the results are reported as follows: no halo (−); weak clearing zone between 1 and 2 mm (+), and well-defined clearing zone larger than 3 mm (++). To determine the lipolytic activity of *Saccharomyces* strains, overnight cultures were streaked in a straight line onto 10% (*v*/*v*) egg yolk agar, as described by Imre et al. [50]. After incubation for 72 h at 37 °C, the precipitation area around the colonies indicated the presence of phospholipase activity.

##### Hemolytic Activity

The hemolytic activity of yeast strains was tested to determine their pathogenicity. Overnight cultures were inoculated onto YPD agar medium supplemented with 7% (*v/v*) human blood (provided by Amar Telidji University for laboratory uses). After 48 h of incubation at 37 °C, hemolysin production was analyzed. Colonies showing transparent halos were classified as β hemolysis (complete hemolysis), while the presence of green or brown halos around colonies was interpreted as α hemolysis (partial hemolysis). No reaction observed around colonies was considered γ hemolysis (no hemolysis) [51].

##### Gelatinase Activity

For gelatinase activity, the assay was performed on YPD agar supplemented with 3% (*w/w*) gelatin. A 0.1 mL aliquot of each overnight culture was inoculated onto the surface of the tested medium and incubated at 37 °C for 24 h. The plates were then refrigerated at 4 °C for 5 h prior to evaluation. The presence of liquefied zones around the colonies indicated gelatin hydrolysis [52].

##### Biogenic Amines Production

The ability to produce biogenic amines (BAs) by yeast strains was evaluated using the method proposed by Staniszewski and Kordowska-Wiater [39]. Yeasts were streaked onto YPD agar plates supplemented with 0.006% bromocresol purple and a mixture of amino acids (1% total mass concentration), including tyrosine, histidine, phenylalanine, leucine, tryptophan, arginine, and lysine in equal ratios. After incubation of plates for 7 days at 37 °C, the observations focused on changes in the medium’s appearance that could indicate the presence of BAs. During the incubation period, the growth of strains and any color changes in the medium were checked daily. It was found that yeasts producing BAs showed a purple halo around the growth area, reflecting a decrease in pH, while those with yellow halos indicated only glucose fermentation.

### 4.4. Statistical Analysis

All statistics and figures were carried out using SigmaPlot for Windows, version 12.0 (Systat Software, San Jose, CA, USA). Each experiment was performed in triplicate and the data are presented as mean values ± standard deviations. The results of bile salts and acidic pH tolerance, auto-aggregation, hydrophobicity, and DPPH reduction were differentiated at *p* < 0.05 using one-way analysis of variance followed by Duncan’s multiple-range tests.

## 5. Conclusions

In conclusion, the present investigation provides new information about the diversity of *Saccharomyces cerevisiae* strains involved in natural fermentations of Algerian traditional food, and their probiotic properties. The molecular identification revealed fifteen *S. cerevisiae* strains, especially obtained from sourdoughs and marinated peppers samples. All tested strains showed high resistance to pH 2.5 and grew well at 0.3% bile salts. The auto-aggregation rates of tested strains remarkably increased with incubation time, while seven of fifteen strains showed high hydrophobicity values in xylene. All *S. cerevisiae* strains showed a good antioxidant activity with DPPH reduction percentages, ranging from 24.29% to 30.29%, and exhibited varying levels of antibacterial activity, but none of these strains inhibited the growth of *L. innocua*. A lack of hemolysis, gelatinase, protease, phospholipase, and biogenic amines production was recorded for all tested strains (with the exception of T27), whereas a strong phytase activity was observed in all strains. Based on these characteristics, five *S. cerevisiae* strains (O08, O12, O17, O20, and T26) showed promising probiotic properties and safety aspects, which can be used as potential probiotic strains. However, deep investigations are needed prior to their applications in the food industry and/or in the medical field, together with evaluation of immune-modulatory activity, antifungal susceptibility, genome-based screening for virulence factors, and bioactive compounds characterization, as well as applications in the food matrix and in in vivo conditions.

## Figures and Tables

**Table 1:** *Saccharomyces cerevisiae* strains categorized by sources with laboratory codes and accession numbers.

Strain Code	Origin	Accession Number *
T22 T25 T27 T07 T12 T15 T20 T26 T05	Sourdough (soft wheat)	PX945044 PX945045 PX945047 PX945049 PX945041 PX945042 PX945043 PX945046 PX945048
P12	Marinated pepper	PX945040
O12 O17 O20 O08 O05	Sourdough (barley)	PX945035 PX945036 PX945037 PX945039 PX945038

* The accession numbers of D1/D2 domain of LSU sequences deposited in GenBank.

**Table 2:** Sugar profile of *Saccharomyces cerevisiae* strains.

Strains	Lactose		Sucrose		Arabinose		Maltose		Galactose		Xylose		Glucose	
	Ass *	Fer **	Ass	Fer	Ass	Fer	Ass	Fer	Ass	Fer	Ass	Fer	Ass	Fer
*S. cerevisiae* O12	−	−	++	++	−	−	++	++	+	−	−	−	+	++
*S. cerevisiae* O17	−	−	++	++	−	−	++	++	+	−	−	−	+	++
*S. cerevisiae* O20	−	−	++	++	−	−	+	w	+	++	−	−	+	++
*S. cerevisiae* T07	−	−	++	++	−	−	++	++	++	++	−	−	++	++
*S. cerevisiae* T12	−	−	++	++	−	−	++	++	++	++	−	−	++	++
*S. cerevisiae* T15	−	−	++	++	−	−	++	++	++	++	−	−	++	++
*S. cerevisiae* T20	−	−	++	++	−	−	++	++	+	−	−	−	++	++
*S. cerevisiae* P12	−	−	−	−	−	−	−	−	−	−	−	−	+	+
*S. cerevisiae* T22	−	−	++	++	−	−	++	++	+	−	−	−	++	++
*S. cerevisiae* T25	−	−	++	++	−	−	++	++	++	++	−	−	++	++
*S. cerevisiae* T27	−	−	++	++	−	−	++	++	++	++	−	−	++	++
*S. cerevisiae* O08	−	−	++	++	−	−	+	++	+	−	−	−	+	++
*S. cerevisiae* T05	−	−	++	++	−	−	++	++	+	−	−	−	+	++
*S. cerevisiae* O05	−	−	++	++	−	−	+	++	+	−	−	−	+	++
*S. cerevisiae* T26	−	−	++	++	−	−	++	++	+	−	−	−	++	++

* Ass: Assimilation; ** Fer: Fermentation; −: Negative reaction; w: Weak reaction; +: Moderate reaction; ++: Strong reaction.

**Table 3:** Antibacterial activity of *S. cerevisiae* strains against *E. coli*, *S. aureus*, and *L. innocua.*

Strains	*E. coli* ATCC 25922	*S. aureus* ATCC 29213	*L. innocua* ATCC 33090
O12	11 *	7	−
O17	−	12	−
O20	6	7	−
T07	6.5	6	−
T12	6	6.5	−
T15	6	6	−
T20	6	6	−
P12	12.5	7	−
T22	13	−	−
T25	−	6.5	−
T27	6.5	6	−
O08	−	12	−
T05	7	7	−
O05	6	11	−
T26	11.5	−	−

* Mean value in millimeter (*n* = 2); −, no inhibition (zone ≤ 5 mm); weak inhibition (zones 6–10 mm); moderate inhibition (zones 11–15 mm).

**Table 4:** Enzymatic activity and safety aspects of *S. cerevisiae* strains.

Strains	Enzymatic Activities				
	Phytase	Protease	Phospholipase	Gelatinase	Hemolysin
*S. cerevisiae* O12	28 *	−	−	−	γ
*S. cerevisiae* O17	31	−	−	−	γ
*S. cerevisiae* O20	32	−	−	−	γ
*S. cerevisiae* T07	26	−	−	−	γ
*S. cerevisiae* T12	27	−	−	−	γ
*S. cerevisiae* T15	28	−	−	−	γ
*S. cerevisiae* T20	30	−	−	−	γ
*S. cerevisiae* P12	25	−	−	−	γ
*S. cerevisiae* T22	30	−	−	−	γ
*S. cerevisiae* T25	28	−	−	−	γ
*S. cerevisiae* T27	26	−	−	−	γ
*S. cerevisiae* O08	28	−	−	−	γ
*S. cerevisiae* T05	27	−	−	−	γ
*S. cerevisiae* O05	28	−	−	−	γ
*S. cerevisiae* T26	27	−	−	−	γ

* Diameter [mm]; Positive reaction halos ≥ 20 mm; −: Negative reaction; γ: non-hemolysis.

## Data Availability

Data will be made available on request.

## Supplementary Materials

The following supporting information can be downloaded at: https://www.mdpi.com/article/10.3390/molecules31142440/s1, Table S1. Gastrointestinal stress tolerance of *Saccharomyces cerevisiae* strains: growth in presence of 0.3% bile salts., Table S2. Gastrointestinal stress tolerance of *Saccharomyces cerevisiae* strains: growth under acidic conditions (pH 2.5) at 37 °C
