# Supplementary material for: In Vitro Study of Probiotic Properties and Safety Aspects of Saccharomyces Yeast Strains Isolated from Traditional Fermented Food in Algeria
Source: Molecules. 2026 Jul 12;31(14):2440. doi: 10.3390/molecules31142440 (PMC13414238; doi:10.3390/molecules31142440)
Supplement: Supplementary file 1 [file molecules-31-02440-s001.zip › molecules-4377679-supplementary.pdf]

Table S1. Gastrointestinal stress tolerance of *Saccharomyces cerevisiae* strains: growth in presence of 0.3% bile salts.

Strains	OD_{600}/0h	OD_{600}/4h	OD_{600}/8h	OD_{600}/12h	OD_{600}/16h	OD_{600}/20h	OD_{600}/24h
Saccharomyces cerevisiae O12	0.109±0.004^{xa}	0.358±0.0530^{cd}	0.659±0.059^{de}	1.194±0.051^{b}	1.730±0.042^{a}	1.815±0.035^{a}	1.635±0.025^{a}
Saccharomyces cerevisiae O17	0.109±0.004^{a}	0.247±0.019^{f}	0.600±0.212^{defg}	1.081±0.149^{c}	1.563±0.086^{de}	1.700±0.000^{bc}	1.560±0.013^{bc}
Saccharomyces cerevisiae O20	0.109±0.004^{a}	0.281±0.021^{ef}	0.305±0.006^{j}	1.003±0.003^{cd}	1.700±0.000^{ab}	1.745±0.007^{ab}	1.607±0.009^{ab}
Saccharomyces cerevisiae T07	0.109±0.004^{a}	0.293±0.014^{def}	0.472±0.055^{gh}	0.773±0.069^{f}	1.074±0.083^{i}	1.254±0.058^{e}	1.432±0.028^{g}
Saccharomyces cerevisiae T12	0.109±0.004^{a}	0.284±0.015^{ef}	0.625±0.035^{def}	0.984±0.017^{cd}	1.344±0.000^{g}	1.333±0.039^{e}	1.489±0.027^{defg}
Saccharomyces cerevisiae T15	0.109±0.004^{a}	0.335±0.000^{cde}	0.718±0.000^{cd}	0.933±0.028^{de}	1.147±0.057^{hi}	1.334±0.128^{e}	1.502±0.065^{cdef}
Saccharomyces cerevisiae T20	0.109±0.004^{a}	0.452±0.028^{b}	0.945±0.092^{b}	1.260±0.064^{b}	1.575±0.035^{cd}	1.612±0.017^{c}	1.489±0.015^{defg}
Saccharomyces cerevisiae P12	0.109±0.004^{a}	0.252±0.045^{f}	0.501±0.001^{fgh}	0.993±0.017^{cd}	1.486±0.033^{ef}	1.669±0.004^{bc}	1.449±0.018^{fg}
Saccharomyces cerevisiae T22	0.109±0.004^{a}	0.400±0.028^{bc}	1.013±0.004^{ab}	1.227±0.008^{b}	1.442±0.012^{f}	1.648±0.025^{bc}	1.474±0.001^{efg}
Saccharomyces cerevisiae T25	0.109±0.004^{a}	0.287±0.024^{ef}	0.579±0.040^{efgh}	0.848±0.056^{ef}	1.118±0.071^{i}	1.511±0.016^{d}	1.541±0.014^{cd}
Saccharomyces cerevisiae T27	0.109±0.004^{a}	0.230±0.013^{f}	0.493±0.024^{gh}	0.857±0.030^{ef}	1.220±0.035^{h}	1.445±0.021^{d}	1.522±0.009^{cde}
Saccharomyces cerevisiae O08	0.109±0.004^{a}	0.265±0.007^{f}	0.45±0.071^{hi}	1.043±0.053^{c}	1.635±0.035^{bcd}	1.750±0.028^{ab}	1.540±0.071^{cd}
Saccharomyces cerevisiae T05	0.109±0.004^{a}	0.398±0.011^{bc}	0.355±0.064^{ij}	0.839±0.034^{ef}	1.322±0.004^{g}	1.6575±0.011^{bc}	1.521±0.039^{cde}
Saccharomyces cerevisiae O05	0.109±0.004^{a}	0.324±0.035^{def}	0.810±0.042^{c}	1.180±0.057^{b}	1.550±0.071^{de}	1.607±0.151^{c}	1.601±0.001^{ab}
Saccharomyces cerevisiae T26	0.109±0.004^{a}	0.777±0.097^{a}	1.127±0.005^{a}	1.388±0.013^{a}	1.650±0.021^{abc}	1.615±0.007^{c}	1.535±0.026^{cd}

x Mean value (n = 3) ± Standard deviation. Different lowercase letters (a–j) in the same column indicate significant differences ($P < 0.05$) in growth among *Saccharomyces cerevisiae* strains

Table S2. Gastrointestinal stress tolerance of *Saccharomyces cerevisiae* strains: growth under acidic conditions (pH 2.5) at 37 °C.

Strains	OD_{600}/0h	OD_{600}/4h	OD_{600}/8h	OD_{600}/12h	OD_{600}/16h	OD_{600}/20h	OD_{600}/24h
Saccharomyces cerevisiae O12	0.107±0.002^{xa}	0.290±0.012^{f}	0.916±0.015^{f}	1.100±0.023^{e}	1.283±0.030^{d}	1.524±0.000^{d}	1.651± 0.021^{e}
Saccharomyces cerevisiae O17	0.107±0.002^{a}	0.335±0.025^{e}	0.880±0.005^{g}	1.155±0.008^{c}	1.431±0.010^{b}	1.680±0.009^{b}	1.666±0.004^{e}
Saccharomyces cerevisiae O20	0.107±0.002^{a}	0.237±0.012^{g}	0.967±0.002^{e}	1.123±0.013^{d}	1.278±0.025^{d}	1.589±0.054^{c}	1.734± 0.037^{cd}
Saccharomyces cerevisiae T07	0.107±0.002^{a}	0.324±0.002^{ef}	0.566±0.003^{i}	0.660±0.003^{g}	0.755±0.004^{f}	0.792±0.023^{f}	0.860±0.009^{f}
Saccharomyces cerevisiae T12	0.107±0.002^{a}	0.287±0.009^{f}	0.384±0.014^{j}	0.432±0.017^{h}	0.479±0.021^{g}	0.498±0.013^{g}	0.524±0.003^{g}
Saccharomyces cerevisiae T15	0.107±0.002^{a}	0.248±0.000^{g}	0.308±0.003^{k}	0.350±0.008^{i}	0.393±0.013^{h}	0.427±0.012^{h}	0.560±0.004^{g}
Saccharomyces cerevisiae T20	0.107±0.002^{a}	0.602±0.016^{a}	1.178±0.010^{a}	1.306±0.017^{a}	1.434±0.025^{b}	1.665±0.004^{b}	1.834±0.031^{a}
Saccharomyces cerevisiae P12	0.107±0.002^{a}	0.412±0.075^{bc}	0.962±0.008^{e}	1.059±0.011^{f}	1.157±0.013^{e}	1.618±0.008^{c}	1.665±0.012^{e}
Saccharomyces cerevisiae T22	0.107±0.002^{a}	0.413±0.030^{bc}	1.118±0.004^{b}	1.272±0.003^{b}	1.427±0.002^{b}	1.717±0.025^{a}	1.803±0.025^{ab}
Saccharomyces cerevisiae T25	0.107±0.002^{a}	0.208±0.000^{g}	0.316±0.003^{k}	0.336±0.004^{i}	0.357±0.005^{i}	0.412±0.008^{h}	0.450±0.000^{h}
Saccharomyces cerevisiae T27	0.107±0.002^{a}	0.221±0.010^{g}	0.386±0.033^{j}	0.357±0.023^{i}	0.329±0.014^{j}	0.360±0.000^{i}	0.382±0.001^{i}
Saccharomyces cerevisiae O08	0.107±0.002^{a}	0.423±0.004^{b}	0.997±0.009^{d}	1.169±0.008^{c}	1.342±0.008^{c}	1.601±0.003^{c}	1.819±0.049^{ab}
Saccharomyces cerevisiae T05	0.107±0.002^{a}	0.379±0.007^{cd}	1.002±0.000^{d}	1.083±0.001^{e}	1.165±0.001^{e}	1.468±0.006^{e}	1.816±0.058^{ab}
Saccharomyces cerevisiae O05	0.107±0.002^{a}	0.323±0.014^{ef}	0.847±0.013^{h}	1.157±0.016^{c}	1.467±0.020^{a}	1.742±0.045^{a}	1.721±0.006^{d}
Saccharomyces cerevisiae T26	0.107±0.002^{a}	0.347±0.005^{de}	1.058±0.020^{c}	1.172±0.019^{c}	1.287±0.019^{d}	1.595±0.021^{c}	1.776±0.026^{bc}

x Mean value (n = 3) ± Standard deviation. Different lowercase letters (a–k) in the same column indicate significant differences ($P < 0.05$) in growth among *Saccharomyces cerevisiae* strains
